# The Tullio effect in a patient qualified for cochlear implantation: Diagnosis, management and rehabilitation performance: A case report

**DOI:** 10.1097/MD.0000000000031867

**Published:** 2022-11-18

**Authors:** Katarzyna Amernik, Renata Twardowska, Ewa Jaworowska

**Affiliations:** a Department of Otolaryngology, Pomeranian Medical University, Szczecin, Poland; b Wolters Kluwer, Mumbai, Maharashtra, India.

**Keywords:** cochlear implant, hearing loss, perilymphatic fistula, the Tullio effect, vertigo

## Abstract

A case study on the qualification and treatment of profound hearing loss with a cochlear implant in a patient with a positive Tullio effect is described. To our knowledge this is the first such case reported in the literature.

**Patient concerns:**

A 46-year-old woman was admitted due to sudden hearing loss in the right ear (RE). The patient had suffered from bilateral hearing loss since childhood and was fitted with hearing aids on the RE successfully, in contrast to the left ear. While undergoing pure-tone audiometry, a positive Tullio effect was observed in the RE. The average hearing threshold for the RE was 95 dB. Due to the lack of effective treatment for sudden hearing loss, the patient was qualified for cochlear implantation. The patient’s attempts to place a hearing aid on the RE resulted in dizziness.

**Diagnoses:**

Computed tomography excluded the presence of a perilymphatic fistula, which could have been the cause of the patient’s vertigo.

**Interventions:**

During the surgical procedure of cochlear implantation, considering the possible mechanisms of the Tullio effect, the incus was removed and the niche of the oval window was filled with fragments of connective tissue. The postoperative course was uneventful.

**Outcomes:**

Three months after implantation, speech intelligibility in the free field was 80% of the correctly repeated elements of the numerical test, at 65 dB sound pressure level. An acoustic stimulation test was performed during tonal audiometry and no preexisting symptoms were observed.

**Lessons:**

A positive Tullio effect does not contraindicate treating hearing loss by means of cochlear implantation. When the Tullio effect is present, it is necessary to exclude presence of perilymphatic fistula. During cochlear implant surgery, in a patient with a positive Tullio effect, it is reasonable to disconnect the ossicular chain with the simultaneous sealing of the oval window niche.

## 1. Introduction

The Tullio effect was first described in 1929 by Italian biologist Pietro Tullio, as an acoustically induced imbalance in pigeons with a previously performed fistula on the semicircular canal.^[1]^ In the following years it was discovered that the Tulio effect can also be induced in patients without a perilymphatic fistula, however the mechanism of this variant of the Tulio effect is not precisely understood.^[2,3]^ One of the first explanations for this sound-induced vertigo was given by Merchant and Schuknecht^[3]^ who saw it as vestibular atelectasis, with its characteristic collapse of the walls of the ampulla and utricula. Merchant and Schuknecht outlined the pathological connection of these structures with the stapes footplate, which leads to abnormal excitation of the organ balance during acoustic stimulation.

Here, we present a case study of a Tullio – positive patient who was eligible for treatment of hearing loss with a cochlear implantation. According to our knowledge, this is the first report in the literature.

## 2. Case description

A 46-year-old woman was admitted to the Department of Otolaryngology with vertigo and sudden sensorineural hearing loss (SNHL) in her right ear (RE). In the past, the patient was rehabilitated for bilateral SNHL with hearing aids for RE, the left ear (LE) had not been treated since childhood due to lack of benefit from rehabilitation on this side. Hearing loss was diagnosed during childhood, probably after streptomycin treatment. On admission, there was no spontaneous nystagmus, the Romberg test result was normal, and a rightward stagger was observed in the walking test. During the RE testing, especially in the low tone range (250–500 Hz), the patient complained of a feeling of dizziness and nausea, accompanied by right-sided nystagmus. No spontaneous nystagmus was observed on videonystagmography, and normal bilateral vestibular excitability was confirmed by caloric tests. The patient had bilateral SNHL, severe on the RE and profound hearing loss in the LE, results are shown in Table 1.

**Table 1:** The patient’s audiometry for RE.

	125 Hz	250 Hz	500 Hz	1 kHz	2 kHz	3 kHz	4 kHz
2009*	60 dB	65 dB	75 dB	110 dB	UMA	UMA	UMA
2021** SSNL	95 dB	100 dB	100 dB	115 dB	UMA	UMA	UMA
2021***	85 dB	90 dB	95 dB	115 dB	UMA	UMA	UMA

* Baseline audiometry.

** Audiometry on onset of sudden hearing loss.

*** 2 months after conservative treatment of SNHL.

UMA = unmarkable.

Impedance audiometry showed bilateral type A tympanogram, absence of stapedius reflex, and normal pressure and compliance values.

Auditory brainstem evoked potentials of 90 dB and 100 dB nHL to click type recorded responses with normal morphology.

Conservative treatment with intravenous dexamethasone was administered but no hearing improvement was achieved. The detailed results of PTA is shown in Table 1.

An initial qualification for cochlear implantation for the RE was performed. Computed tomography showed no perilymphatic fistula and magnetic resonance imaging displayed no abnormalities. After logopedic and psychological consultations, the patient was qualified for cochlear implantation in the RE. The patient’s great need for communication at work and in everyday life prompted her to try to change the settings of her current hearing aid in the RE while awaiting surgical treatment. However, attempts to adjust the hearing aids have resulted in dizziness and nausea. The dizziness continued to occur on subsequent attempts to wear the hearing aids, despite intervals of 1 week between fittings.

Cochlear implantation surgery was performed in an ENT Clinic, using a cochlear implant (CI) 632 device. Taking into account the possible mechanisms of the Tullio effect, measures were taken during the surgery; the ossicular chain was disconnected, the incus was removed and the oval window niche was additionally filled with fragments of connective tissue. When the membrane of the round window was opened, no outflow of perilymphs was observed under increased pressure.

**Figure 1.**
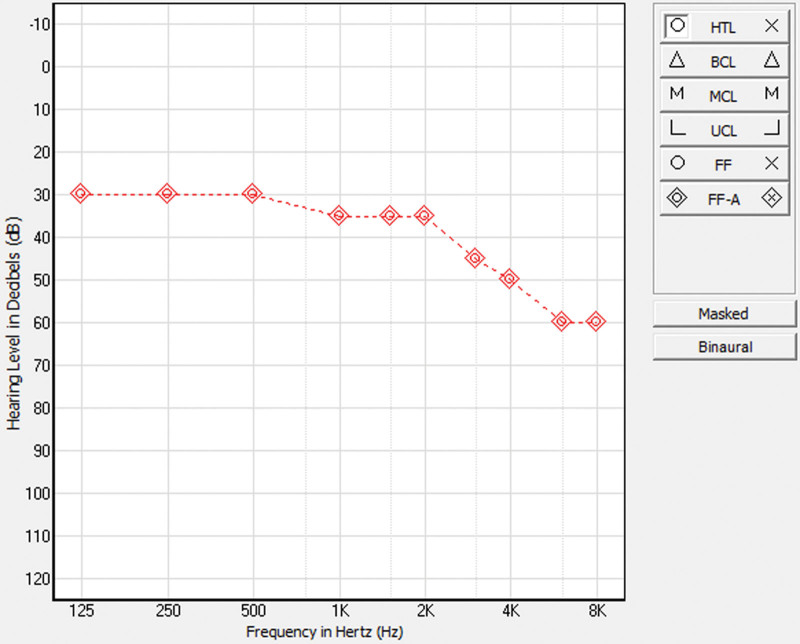
Illustration of the thresholds that were plotted on the audiogram at the time of cochlear implant qualifications.

The postoperative period was uneventful. The Kanso 2 sound processor was activated 4 weeks after surgery. The patient has also been fitted with a hearing aid for the LE, 8 weeks after implantation, but the outcome was not satisfactory. Six months after the activation of the CI processor and the beginning of rehabilitation, the patient did not experience any vertigo during electrical or acoustic stimulation during audiometry tests. The patient currently participates in auditory and speech rehabilitation. As shown in Figure 1, significant progress was made in the free field hearing with the CI processor.

The patient was able to differentiate common phrases without lip reading. According to categories of auditory performance, the patient’s results fulfilled Category 5 criteria.

Speech intelligibility in the free field was 80% of the correctly repeated elements of the numerical test, at 65 dB sound pressure level. The Abbreviated Profile of Hearing Aid Benefit (APHAB) questionnaire was used to assess the effectiveness of the CI matching.

**Figure 2.**
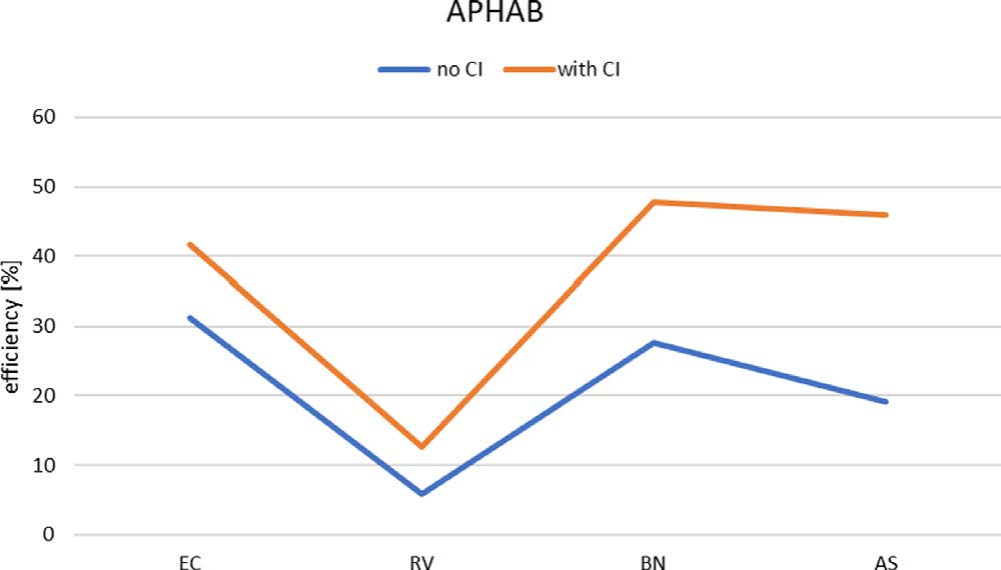
The APHAB test results presented the benefits of using the CI sound processor, CI = cochlear implant, APHAB = Abbreviated Profile of Hearing Aid Benefit.

APHAB^[4]^ is an assessment of the effectiveness of the fitting of a hearing aid or speech processor, based on a questionnaire with 24 questions to determine the ability to communicate in different listening environments: in silence (Ease of communication), in the presence of echo (Reverberation), in the presence of background noise, and the degree of acceptance of unpleasant sounds (Aversiveness of sounds). The results of APHAB summarized in the form of a graph confirm the improvement of the patient’s ability to communicate in different acoustic environments, 6 months after activation of the speech processor (Fig. 2).

## 3. Discussion

The literature reports cases in which Tullio phenomenon happens in healthy individuals with particularly sensitive inner ear receptors^[1]^ and in patients with Menier disease, in patients with a lesion of the stapes and in temporal bone anomalies. Moreover, these problems concerning the ear occur when there are fractures with a fissure running through the vestibulum, displacement of the stapes, vestibular inflammation or a perilymphatic fistula in Lyme disease or congenital syphilis and in children with congenital malformations of the inner ear.^[1]^ The Tullio effect can be induced in the deaf ear, but it can be induced in the ear with a non-active labyrinth, which was demonstrated by Tullio himself by injecting cocaine into the pigeon’s labyrinth, destroying the endings of the nerve fibers. After such destruction it was not possible to induce the described effect.^[1]^

To our knowledge, the problem of qualification for cochlear implantation in a patient with a positive Tullio effect has not yet been described in the literature. In our patient, bilateral profound SNHL had been present since childhood; however due to sudden hearing loss, she lost the use of hearing aids and developed vertigo in the form of positive Tullio effect. This happened when the right ear was either stimulated by hearing aid or by the audiometric examination. Although a perilymphatic fistula could be 1 of the possible mechanisms of these symptoms, it was nonetheless excluded in this patient based on computed tomography. Perilymphatic fistulas on imaging reveal the presence of air in the inner ear structures^[5]^ though they may also be asymptomatic if they involve leakage of perilymph through the round or oval window area. In the case of our patient, this mechanism was also considered by the authors.^[5]^ The appearance of this type of fistula may be responsible for sudden hearing loss with dizziness^[5,6]^ despite the fact that in such a situation 1 of the clinical signs is fluctuating hearing impairment, which was not observed in this case.

The possibility of a window fistula in our patient was taken into account because window fistulas more often give symptoms in the form of balance disorders than in the form of nystagmus.^[6]^ The patient’s history did not suggest such an occurrence (no factor could have caused pressure injury).

The mechanism of the appearance of this phenomenon in our patient—having ruled out chronic middle ear disorders, enlarged vestibular aqueduct syndrome, bone destruction leading to perilymphatic fistula, or chronic Lyme disease^[1]^—can be explained by atelectasia of the vestibulum described by Wenzel.^[3]^ Wenzel et al described 4 patients with impaired vestibular excitability and nystagmus, with vertigo induced by acoustic stimulation as the likely cause of vestibular atelectasia.^[3]^ For many years it was not possible to confirm this suspicion on the basis of imaging studies. Recently, Marc et al pointed to the NMR protocol, where it is possible to demonstrate the collapse of the endolymphatic sac walls; however, in the discussed case no such pathology could be visualized.^[2]^

The literature describes a case of Tullio effect 2 years after cochlear implantation.^[7]^ This patient was surgically treated by disconnecting the ossicular chain. Regarding our patient (after informed consent) the ossicular chain was disconnected with remove to protect the patient from the Tullio effect. Coordes described the possible mechanisms of dizziness after cochlear implant surgery^[8]^ with particular emphasis on vertigo caused by loud sounds, without electrical stimulation of the cochlear implant. In the group of implanted patients studied by Coordes et al 18% of the patients complained of vertigo induced by acoustic stimulation, especially broadband noise. In this study, 9% of patients who qualified for cochlear implantation complained of noise-induced vertigo; unfortunately, the follow-up of these patients was not described and they were excluded from the study.

In this case, after considering the various possible mechanisms of the Tullio effect and the necessity to provide the patient with a cochlear implant, we decided to perform the procedure in a typical way, with simultaneous removal of the incus and intraoperative filling of the niche of the oval window with connective tissue.^[7]^ The electrode was implanted through a round window by sealing its niche. Regardless of the potential mechanism of the Tullio effect, the procedures performed could not adversely affect the symptoms and potentially protected the patient from postoperative dizziness. The observation period in the described case is 6 months. The patient remains under laryngological follow-up procedures, and so far no dizziness has been observed either during electrical or acoustic stimulation. The patient is using the cochlear implant system and making gradual progress in rehabilitation, which proves the effectiveness of the solution used in this case.

## 4. Conclusions

When the Tullio effect is present in a patient with profound hearing loss, it is necessary to perform differential diagnosis between positive fistula sign and the Tullio effect. It is also necessary to exclude the presence of perilymphatic fistula in such cases.

During cochlear implant surgery on a patient with a positive Tullio effect, it is judicious to disconnect the ossicular chain with simultaneous sealing of the oval window niche.

A positive Tullio effect does not contraindicate treating hearing loss by means of cochlear implantation.

## Acknowledgements

We thank Hubert Gostyński and Gary Stewart for providing language help in preparing this article.
